# Assessing the adequacy of funding for robotic-surgery hospital stays: a focused cost analysis based on 1,722 procedures in a French university hospital

**DOI:** 10.1007/s11701-026-03365-x

**Published:** 2026-04-03

**Authors:** Arthur Lacassagne, Solène Molle, Lionel Badet, Pierre-Adrien Bolze, Marc Colombel, Fréderic Hameury, Jean-Michel Maury, Arnaud Pasquer, Charles-André Philip, Alain Ruffion, François Tronc, André Lecoanet, Christell Julien, Xavier Armoiry

**Affiliations:** 1 School of Pharmacy (ISPB), Pharmacy Department, University of Lyon, UMR CNRS 5510 MATEIS, “Edouard Herriot” Hospital, Lyon, France; 2 Direction de l’Ingénierie Biomédicale et de l’Equipement, Hospices Civils de Lyon, Lyon, France; 3 Service d’urologie, Hospices Civils de Lyon, Hôpital Edouard Herriot, Chirurgie de la Transplantation, Lyon, France; 4 Service de Chirurgie Gynécologique, Oncologique et Obstétrique, Hospices Civils de Lyon, Hôpital Lyon sud, Pierre-Bénite, France; 5 Service de Chirurgie Pédiatrique, Hospices Civils de Lyon, Hôpital Femme-Mère Enfant, Bron, France; 6 Service de Chirurgie Thoracique, Transplantation Pulmonaire et Cardio Pulmonaire, Hospices Civils de Lyon, Hôpital Louis Pradel, Bron, France; 7 Service de Chirurgie Viscérale, Digestive et Bariatrique, Hospices Civils de Lyon, Hôpital Edouard Herriot, Lyon, France; 8 Service de Gynécologie-Obstétrique, Hospices Civils de Lyon, Hôpital Femme-Mère Enfant, France, France; 9 Urology Department, Lyon Cancer Innovation Center (EA 3738 CICLY), Lyon Sud Medical School, Lyon Sud Hospital, Hospices Civils de Lyon, University of Lyon 1, Lyon, France; 10 Département d’information médicale, Hospices Civils de Lyon, Pôle de santé Publique, Lyon, France; 11 Pharmacy Department, Edouard Herriot Hospital (Lyon University hospitals), 24 rue Trarieux, Lyon, 69003 France

**Keywords:** Robotic-assisted surgery, Cost, Funding, Benchmark

## Abstract

Context: Robotic-assistance is increasingly used in French hospitals and funded through standard Diagnosis-Related Group (DRG) tariffs. We evaluated the actual costs of robotic-assisted surgical procedures (RASP) and compared these with hospital revenues to assess whether current funding levels are sufficient to cover procedural costs. Materials and methods: This cross-sectional study was conducted in Lyon University Hospitals (France). We examined all RASP performed over January 2023-June 2024. We matched medical device usage data from the DaVinci system manufacturer’s database with the hospital database, calculated related costs, and compared these against the costs/ tariffs of DRGs using the national cost benchmarks. Results: Based on a final sample of 1,722 procedures, we observed substantial cost variations between surgeries for RASP-specific medical devices, with higher-cost procedures such as those requiring staplers/ reloads (mean costs: €2,858 to €3,189) and lower-cost procedures (€1,207 to €1,621). Among the 21 most frequently performed surgeries, the mean length of stay (LoS) was numerically lower than national benchmarks in 20 cases. Fourteen were associated with mean costs of RASP devices exceeding the national benchmark within the same cost category (medical consumables) (range of additional cost: +€309 to +€2,428); incorporating potential savings resulting from reduced LoS could offset these additional costs in three cases. Medical consumable costs were lower for five surgeries (range of cost reduction: -€109 to -€726). Conclusions: In most cases, hospital funding based on current DRG tariffs is insufficient to cover the actual costs of RASP, suggesting that these stays are undervalued even though a reduced LoS may partially offset the additional costs.

**Supplementary Information:**

The online version contains supplementary material available at 10.1007/s11701-026-03365-x.

## Introduction

Since its launch in the late 1990s, the DaVinci surgical system (Intuitive, USA) has become a global benchmark in robotic-assisted surgery [1]. Originally designed to enhance surgical precision and reduce post-operative complications, it is now routinely used across a variety of specialties including urology, gynaecology, thoracic and digestive surgery. In 2022, more than 6,000 Da Vinci robots were reported to be in use worldwide [2], with France ranking as the third largest user country after the United States and Japan [3]. As of November 2025, more than 300 DaVinci robotic systems were installed in French hospitals, with all university hospitals being equipped as well as 39% of private centers with surgical activities eligible for robotic assistance.

Consistent with the growing number of installations in French hospitals, the prevalence of robotic-assisted procedures is also expanding. An observational study reported that among 286,000 surgeries undertaken between March 2020 and June 2022, 18% involved robotic assistance, including 71% in urology, 16% in digestive surgery and 13% in gynaecology [4].

This rapid expansion contrasts with economic concerns associated with the use of robotic-assisted surgery, which remains a source of debate among hospital stakeholders, in a context where French hospitals, especially public ones, are facing a structural crisis marked by budgetary constraints, staff shortages and increasing pressure on the profitability of medical procedures.

Indeed, the purchase cost of such robotic-systems is substantial (€2 million), to which maintenance and procedure costs must be added, particularly for the supply of single-use and reusable medical devices.

As no regulatory framework exists to enable reimbursement for robotic-assisted surgical devices, hospitals adopting this technique fund these costs from their revenues which are primarily derived from hospital tariffs associated with diagnosis-related groups (DRGs) under the activity-based pricing system [5].

Here, we aimed to evaluate the cost of robotic-assisted surgical procedures (RASP) and to compare these costs against the costs and tariffs of DRGs in relation to corresponding hospital stays.

## Methods

### Study type and population

This was a cross-sectional study conducted at Lyon University Hospitals (HCL). With 5,075 beds, HCL is the second-largest teaching hospital in France and is composed of four main hospitals with surgical activities. Over 83,000 surgical procedures were performed in 2024. As of November 2025, HCL is equipped with five Xi Da Vinci systems (Intuitive SAS, Pessac, France) : one in Edouard Herriot Hospital since July 2019 ; one at Croix-Rousse Hospital since February 2023 ; two in Lyon Sud Hospital (transition from one Si to Xi version in January 2023, and one Xi used in clinical practice since late 2022) ; one in Lyon East Hospital since March 2023.

For the purposes of the study, we included all procedures assisted by the DaVinci surgical system between January 2023 and June 2024.

### Measure of cost for surgical procedures

To calculate the cost of surgical procedures, we focused on medical devices specific to the Da Vinci system. We contacted the manufacturer to obtain data on the use of these dedicated medical devices during each RASP. All DaVinci systems have the capability to record and store the use of each instrument docked onto one of the robotic arms and handled remotely by the surgeon.

For the period from January 2023 to June 2024, we received the Intuitive database as an Excel file listing all instruments and their usage associated with the recorded surgical procedures. Single-use medical devices not recorded by the system (arm and column drapes) were manually added to the list, assuming systematic use of four arms during RASP. For each recorded surgery, the file also included the following variables: case number, site, date, surgical specialty, and procedure label.

Based on the resource usage, we calculated a cost per procedure using the corresponding tariffs in place for each reference within our institution as of September 2024. For reusable instruments with a limited number of uses, the tariffs were adjusted according to the maximum number of uses permitted by the manufacturer.

We also calculated the corresponding costs using a discounted tariff based on commercial agreements with our institution. The discount rate, also referred to as End of Year Discount, increases depending on the thresholds of RASP performed per year per robot and on the total value of consumables orders. For simplicity, we applied a fixed discount rate of 5% for all surgeries recorded over the study period.

### Comparison of costs of procedures to the tariffs perceived for hospital stays

To determine the revenues associated with RASP, we retrieved relevant medical information data related to the surgical procedures of interest. We extracted the list of RASP recorded in the operating room data center, “EASILY bloc,” over the same period. This second database was available as an Excel file containing the following variables: date, location, anonymous patient ID, and procedure label.

In France, hospital stays are primarily funded under the activity-based pricing system (T2A). Each stay is categorized according to a system inspired by diagnosis-related groups (DRG) known as “Groupe Homogène de Malades”(GHM). These groups are used to calculate the tariffs of stays.

With our Medical Information Department (DIM), we extracted from the institutional activity database the GHM classification, the length of stay (LoS) and the tariff for each stay corresponding to an included RASP.

We also incorporated data from the National Costing Study (ENC). The ENC is a nationally used framework to evaluate the cost of care production for all DRG types based on a sample of hospitals, both public and private, participating on a voluntary basis.

We used the following publicly available data from 2024 ENC: mean cost of DRG, mean cost of medical consumables (corresponding to non-implantable medical devices like those used in the operating room), and average LoS.

We referred to this repository as the “EASILY bloc” database.

### Treatment of data and statistical analysis

We matched the “INTUITIVE” and “EASILY bloc” databases using common variables, namely the date, location, and procedure label. The labels of surgeries were based on different nomenclatures between the two databases. Given the greater accuracy of the EASILY bloc nomenclature, the latter was retained in our analyses. In cases of discrepancy between the procedure labels in the databases, medical charts were reviewed to determine the actual surgery performed.

Once the unified database was formed, we conducted several quality checks to ensure data consistency and to identify potential outliers.

Costing data were described using mean ± standard deviation, median and interquartile range, and 10th-90th percentiles. Aggregated cost data were sorted by surgical specialty and grouped by procedure.

For procedures performed more than 50 times, except prostatectomy and partial nephrectomy which had been established long before the study began, we compared the costs across six-month intervals to examine potential reductions over time. Given the non-normal distribution of costs, a Kruskal-Wallis test was used.

#### Base case cost comparison

A base case cost comparison was conducted by calculating the difference between the cost of Da Vinci consumables and the cost of medical consumables as observed in the national costing study. For the national benchmarks used as reference values (medical consumables, cost/tariff of DRG), we calculated weighted averages using the exact same case mix of DRGs observed within our dataset.

#### Exploratory cost comparison

We incorporated additional medical resources to explore their impact on results. First, we included the acquisition and maintenance cost associated with the Da Vinci system. To this end, we considered: 1)- an acquisition cost of €2 M, which includes environmental costs to support the installation of the robot in hospitals, amortized over ten years; 2)- an annual cost of maintenance of €180 K applied from Years 3 to 10; 3)- an average of 500 robot-assisted procedures per year; this led to applying an additional €704 per procedure. Second, we estimated the potential savings associated with LoS reduction, defined as the difference between LoS observed at our hospital and the national average, multiplied by a fixed rate of €1,000 per day of LoS saved, consistent with rates measured at our institution.

### Ethics approval

The study did not require ethics approval. Written informed consent from participants was waived according to national regulations on “non-interventional clinical research” (articles L.1121-1 and R.1121-2 of the French Public Health Code).

## Results

### Description of surgeries and medical device use

A total of 1,772 procedures were recorded in the Intuitive database over the study period.

Of these, 11 (0.62%) were excluded due to the absence of any recorded medical devices usage, reflecting cases in which a robotic surgery had been planned but ultimately not performed.

Of the remaining 1,761 procedures, a further 39 were excluded due to unsuccessful matching with the procedures recorded in our EASILY-bloc database, leaving 1,722 (97.1%) procedures for final analysis.

The three most frequently represented surgical specialties were urology surgery (*n* = 595), digestive surgery (*n* = 494), and gynaecological and breast surgery (*n* = 367). Thoracic surgery accounted for 191 cases, while the remaining procedures comprised paediatric surgery (*n* = 49) and Head & Neck Surgery (*n* = 26).

Twenty-one procedure types were performed more than 20 times over the period, representing 1,375 (79.8%) procedures in total (Table 1).

**Table 1:** Cost (expressed as euros) of Da Vinci system consumables medical devices based on the most frequently performed surgeries (*n* > 20)

Surgical specialty	Types of surgery	*N*	Mean	Standard deviation	Median	Min	Max	10th	25th	75th	90th
Digestive surgery	Proctectomy	69	1,854	454	1,841	1,060	2,897	1,075	1,656	2,018	2,422
	Hepatectomy/ hepatic resection	68	1,950	609	1,998	1,060	3,659	1,060	1,516	2,338	2,741
	Colectomy	48	2,086	598	2,238	1,060	2,913	1,075	1,663	2,507	2,913
	Sleeve Gastrectomy	44	2,858	390	2,881	1,268	3,904	2,541	2,711	3,088	3,265
	Hernia cure	40	1,235	191	1,127	950	1,824	1,109	1,127	1,237	1,482
	Rectopexy	35	1,470	268	1,396	1,219	2,428	1,237	1,396	1,396	1,977
	Ventral hernia repair	33	1,207	309	1,127	950	2,731	950	1,109	1,237	1,385
	Bypass/Transit bipartition	25	3,189	572	3,303	1,280	4,253	2,612	2,786	3,511	3,698
Gynecology and breast surgery	Hysterectomy	198	1,357	193	1,354	950	2,410	1,219	1,237	1,354	1,623
	Myomectomy	65	1,455	153	1,488	1,219	2,002	1,219	1,354	1,506	1,623
	Deep infiltrating endometriosis surgery	22	1,580	558	1,458	925	3,517	1,085	1,354	1,623	2,267
Thoracic surgery	Lobectomy	108	2,932	664	2,932	1,050	5,020	2,218	2,634	3,244	3,620
	Segmentectomy	52	3,154	777	3,064	1,320	4,817	2,219	2,634	3,846	4,222
Urology	Prostatectomy	258	1,282	168	1,219	1,085	1,819	1,109	1,109	1,403	1,416
	Partial nephrectomy	143	1,387	213	1,403	1,060	2,441	1,219	1,268	1,403	1,780
	Renovascular surgery	37	1,488	294	1,403	950	2,598	1,403	1,403	1,403	1,819
	Cystectomy (+ bricker or ureterostomy)	33	2,201	721	2,010	1,060	3,747	1,378	1,800	2,620	3,371
	Enlarged nephrectomy	28	1,259	205	1,244	1,060	1,825	1,085	1,085	1,403	1,621
	Surgery for urological malformations	26	1,397	204	1,400	1,085	2,097	1,244	1,244	1,403	1,645
	Surgery for urinary continence and pelvic organ prolapse	22	1,334	109	1,378	1,035	1,403	1,147	1,354	1,391	1,403
	Cystectomy (+ bladder augmentation, neobladder, bladder replacement, ileal reservoir, or ileocecal bladder)	21	1,621	424	1,378	1,085	2,510	1,219	1,378	1,959	2,216

**Table 2:** Cost (expressed as euros) of DaVinci system medical devices, cost of medical consumables as measured within the national costing study, costs and tariffs of DRGs, length of stay within the hospital, and national length of stay, based on the most frequently performed surgeries (*n* > 20)

Specialty (bold)/ Types of surgery	*N*	(1): Mean cost of DaVinci system medical devices	(2): Benchmark costs (€), i.e. medical consumables	(3): Mean costof DRG (based on national benchmark)	(4): Mean Perceived DRG Tariff	Mean length of stay (days)	Benchmark length of stay (days)	Base case cost comparison $\:\left(1\right)-\left(2\right)$	$\:\frac{\left(1\right)}{\left(3\right)}$	$\:\frac{\left(2\right)}{\left(3\right)}$	$\:\frac{\left(1\right)}{\left(4\right)}$	$\:\frac{\left(2\right)}{\left(4\right)}$
**Digestive surgery**	**494**											
Proctectomy	69	1,854	2,346	18,851	14,241	10.43	15.11	-492	10%	12%	13%	16%
Hepatectomy/ hepatic resection	68	1,950	1,921	14,974	15,120	7.75	10.47	29	13%	13%	13%	13%
Colectomy	48	2,086	1,123	10,768	9,710	6.31	8.59	963	19%	10%	21%	12%
Sleeve Gastrectomy	44	2,858	799	5,385	6,183	2.48	3.44	2,059	53%	15%	46%	13%
Hernia cure	40	1,235	429	3,593	3,178	2.45	1.92	806	34%	12%	39%	13%
Rectopexy	35	1,470	496	4,617	4,303	1.4	3.58	974	32%	11%	34%	12%
Ventral hernia repair	33	1,207	496	4,672	3,703	2.55	3.51	711	26%	11%	33%	13%
Bypass/Transit bipartition	25	3,189	761	5,063	5,651	2.2	3.02	2,428	63%	15%	56%	13%
**Gynecology and breast surgery**	367											
Hysterectomy	198	1,357	725	5,480	4,641	1.75	2.55	632	25%	13%	29%	16%
Myomectomy	65	1,455	547	4,135	3,084	1.34	2.51	908	35%	13%	47%	18%
Deep infiltrating endometriosis surgery	22	1,580	762	5,906	4,797	3.32	3.92	818	27%	13%	33%	16%
**Thoracic surgery**	**191**											
Lobectomy	108	2,932	1,430	11,158	10,392	6.64	7.33	1,502	26%	13%	28%	14%
Segmentectomy	52	3,154	1,362	10,086	9,900	5.67	6.36	1,792	31%	14%	32%	14%
**Urology**	**595**											
Prostatectomy	258	1,282	1,975	8,601	7,029	1.4	3.33	-693	15%	23%	18%	28%
Partial nephrectomy	143	1,387	1,370	10,029	9,529	3.31	5.25	17	14%	14%	15%	14%
Renovascular surgery	37	1,488	776	7,608	4,263	3.97	4.73	712	20%	10%	35%	18%
Cystectomy (+ bricker or ureterostomy)	33	2,201	2,310	20,985	16,683	13.94	17.48	-109	10%	11%	13%	14%
Enlarged nephrectomy	28	1,259	1,423	10,549	10,973	5.79	6.34	-164	12%	13%	11%	13%
Surgery for urological malformations	26	1,397	1,088	7,765	6,151	2.4	4.04	309	18%	14%	23%	18%
Surgery for urinary continence and pelvic organ prolapse	22	1,334	543	5,459	3,987	2.18	3.25	791	24%	10%	33%	14%
Cystectomy (+ bladder augmentation, neobladder, bladder replacement, ileal reservoir, or ileocecal bladder)	21	1,621	2,347	22,146	16,956	15	18.66	-726	7%	11%	10%	14%
**Pediatric surgery**	**49**	**1**,**398**	**787**	**6**,**254**	**5**,**805**	**2.47**	**3.74**	**610**	**22%**	**13%**	**24%**	**14%**

Almost all procedures (98.7%) were undertaken with a DaVinci system version Xi. The remaining ones (1.3%) were performed with the Si version which was still in use at the beginning of the study period.

Among reusable instruments, monopolar curved scissors (88.5% of cases), and bipolar forceps whether fenestrated or Maryland (> 100% of cases) were used in nearly all procedures (supplementary Tables 1 and 2). The same applied to needle drivers (96.9% of cases) with substantial variations across specialties. In 82.4% of cases, a DaVinci forceps was used consistent with the availability of the four-arm robotic-assistance across our hospitals.

Regarding single-use medical devices, aside from arm and column drapes, which were systematically used, no consistent pattern of device use was observed across procedures.

The undiscounted cost of DaVinci system medical devices was €1,723 and after applying the 5% discount rate, the mean cost was €1,637. These values should be seen as purely informative as they are heavily dependent on the case-mix of surgeries performed within each hospital. Indeed, there were substantial variations by surgical specialty and types of surgery.

### Cost analysis by surgery type

The costs reported in this section are undiscounted.

The mean costs for prostatectomy and partial nephrectomy, which were among the most frequently performed procedures (*n* = 258 and *n* = 143 respectively), were €1,282 ± 168 and €1,387 ± 213 respectively, with median [interquartile range] of €1,219 [1,109-1,403] and €1,403 [1,268-1,403] reflecting consistent and standardized instrumentation practices. The main sources of cost variation were the number of needle drivers used (1 vs. 2) and the use of a DaVinci forceps (i.e. Prograsp, Cadiere) in line with the use of three or four robotic arms.

Other moderately expensive procedures were gynaecological surgeries, with mean costs varying between €1,357 and €1,580, and some in digestive surgery, such as hernia and ventral repair with mean costs varying between €1,235 and €1,207 respectively, yet very little variation was observed across similar surgeries as illustrated by interquartile ranges of approximately €110 to €150.

Higher costs procedures were noted in digestive surgery, with mean costs of €1,854 ± 454 for proctectomy, €1,950 ± 609 for hepatectomy/hepatic resection, and €2,086 ± 598 for colectomy, as well as in urology with mean costs for cystectomy with Bricker derivation of €2,201 ± 721.

Compared to the first group, these surgeries incurred higher costs due to the use of an additional instrument from the Da Vinci Energy product line such as the SynchroSeal^®^ in hepatectomy/hepatic resection, or the Vessel Sealer Extend^®^ in colectomy, and/or the use of the Sureform^®^ staplers and their reloads. The standard deviations indicated substantial cost heterogeneity, primarily driven by the number of stapler reloads used.

A third group of surgeries associated with higher costs was identified namely bariatric surgeries (sleeve gastrectomy and bypass/transit bipartition), thoracic lobectomy and thoracic segmentectomy with mean costs of €2,858 ± 390, €3,189 ± 572, €2,932 ± 664, and €3,154 ± 777 respectively, resulting from a systematic use of staplers and reloads, the quantities of which also contributed to the observed cost heterogeneity. For example, among lobectomies, the number of stapler reloads which were used was < 5, 5 to 10, 11 to 16, and > 16 in 11%, 43%, 40%, and 6% of procedures, respectively.

The comparison of costs across six-month intervals for six surgery types did not suggest any reductions in costs over time (supplementary Table 3).

### Comparison of costs for DaVinci system related devices to hospital stays costs and tariffs

#### Base case cost comparison

Among the 21 most frequently performed surgeries, 14 were associated with mean DaVinci system device costs exceeding the national benchmark within the same cost category (medical consumables), with additional costs ranging from €309 (surgeries for urological malformations) to over €2,000 (bypass/transit bipartition: +€2,428; sleeve gastrectomy: +€2,059). For sleeve gastrectomy, the cost of Da Vinci system medical devices represented 53% of the mean cost of hospital stay based on national benchmark, compared with 15% for the same cost category in the ENC.

Two procedure types (hepatic resection and partial nephrectomy) were associated with Da Vinci device costs that were consistent with national benchmark (cost difference of +€29 and +€17 respectively).

For five surgeries, the mean Da Vinci medical devices costs were lower than national means for similar DRG types, with a reduction of cost exceeding €400 for three types of surgery (cystectomy with urinary reconstruction: - €726; prostatectomy: -€693; proctectomy: -€492).

Across all surgical procedures, the mean cost of Da Vinci specific instruments (€1,723) accounted for 18.4% of the weighted mean DRG cost and 20.9% of the weighted mean DRG tariff, compared with 13.7% and 15.7% respectively for the mean cost of medical consumables (€1,297) as reported in the ENC. This indicates a mean incremental cost of €426 relative to the national benchmark. As noted above, these average figures, which are heavily dependent on the case mix of surgeries, are provided for completeness only.

#### Exploratory cost comparison

For hospital stays corresponding to the most frequently performed surgeries (*n* = 21), all but one were associated with numerically lower LoS than national benchmarks. The difference in LoS exceeded one day for 11 types of procedure and was more than two days for six types.

After accounting for acquisition and maintenance costs of the Da Vinci system, and potential savings resulting from reduced LoS, 11 procedures still incurred additional costs compared to the national benchmark (ranging from +€425 to +€2,312), while nine procedures showed negative incremental costs beyond -€100, with savings exceeding €1,500 for five of them (supplementary Table 4).

## Discussion

To our knowledge, this is the first cost estimation of RASP using a large dataset of procedures, both in terms of quantity and diversity, from the French healthcare perspective.

We also compared these costs with the associated DRG tariffs for robotic-assisted surgery.

Our findings indicate that, in most cases, RASP incurs significantly higher medical device costs, both for reusable and single-use instruments, exceeding the average cost of medical consumables, as benchmarked against the ENC.

Several factors may account for these cost differences.

One key reason is the methodology of the ENC, which relies annually on voluntary participation from both public and private hospitals to document DRG costs.

The ENC published in 2025 [6] included data from 96 hospitals (48 from each sector). Of the 48 healthcare facilities from the public hospitals, six were university hospitals and contributed to 43.1% of non-dialysis stays in the public sector sample, which is substantial. However, since the DRGs for robotic-surgery are not specific, it is likely that the cost of these DRGs were calculated by aggregating data from hospitals where surgeries were undertaken with or without robotic-surgery depending on the availability of these systems. This can generate a possible “watering down” of the additional cost associated with Da Vinci system medical devices. The unequal use of robotic-assistance was also highlighted in a 2024 report from the French Academy of Surgery on robotic-assisted surgery activities in France in 2022 which used the French national hospital registry (Programme de Médicalisation des Systèmes d’Information) [7]. The authors showed that robotic activity only represented 15.6% of stays for the procedures selected for the study and that the territorial deployment of robotics was not uniform, either accounting the volume of stays, or the distribution between the different surgical specialties. For instance, the use of robotic-assistance only represented 16.3% and 5.2% of all thoracic and bariatric surgeries respectively in 2022, these surgery specialties being two examples where we identified the greatest difference between the cost of Da Vinci system devices and the national benchmark.

A second explanation may be related to the method that is commonly implemented to calculate the cost of medical device use for hospitals participating in the ENC. When healthcare institutions cannot precisely track, for each hospital stay, the costs associated with the medical consumables used during the stay, the mean cost of medical devices by hospital stay is calculated dividing the sum of all expenses of medical devices by the number of stays. For RASP, this average is derived by summing all Da Vinci system device costs over a year and dividing by the number of robotic surgeries performed. As a result, each DRG cost incorporates the same average, regardless of the actual costs incurred for individual procedures. Over our study period, this method yields an average Da Vinci system device cost of €1,723, uniformly applied to all robotic-surgery DRGs, effectively ignoring the true variability in individual procedure costs by surgical specialty.

In our analysis, we categorised surgeries by Da Vinci system device costs into three groups: « least expensive », « moderately expensive », « most expensive ». We showed that cost differences were primarily driven by the use of staplers and reloads. This observation is expected as these procedures would require the use of stapler and reloads regardless of robotic assistance. Of note, we verified that the cost of Da Vinci system stapler and reloads is comparable to those from other brands, used in laparoscopic stapling.

Our results indicate that the additional costs of robotic-assisted surgery primarily result from dedicated-surgical instruments such as monopolar curved scissors, bipolar forceps, needle drivers, and other forceps.

It should be noted that the study period included the phases of learning curve for some procedures as the Da Vinci Xi systems were installed in two of the four hospitals during year 2023. While we did not identify notable difference on costs after dividing the study period by six-months intervals, we assume that some cost estimates are not stabilised since surgeons may have initially used expensive devices occasionally at the beginning of the learning curve and later discontinued their use. Similarly, as surgeons gain experience in robotic surgery, changes in instrument utilisation may occur, potentially leading to reduced costs. For example, in a study of > 1,000 adult patients who underwent robotic thoracic surgery (mainly lobectomies and segmentectomies) within a high-volume single center, Durand et al. reported a technique of vascular sewing and knotting which was preferred over automatic stapling [8], meaning that the corresponding costs should be dramatically reduced compared to our practice.

Last, the surgical case mix observed during the study period may have included procedures selected as part of the learning curve and therefore may not accurately reflect the true patient recruitment within our university hospitals. For example, in our RASP cohort, sleeve gastrectomy may have been overrepresented relative to revisional bariatric procedures.

We showed that sleeve gastrectomy was associated with one of the largest proportion of Da Vinci system devices relative to the cost of DRG or its tariff. In a systematic review that selected 14 retrospective studies comparing laparoscopic to robotic-assisted bariatric surgery, Affolter et al. [9] showed that total hospital costs favoured laparoscopic surgery with an absolute difference of USD 3,819. A slight advantage of robotic surgery was reported with regards to hospital stay and complication rates. Concerning the LoS, our results suggest a similar trend compared to national benchmark.

Affolter et al. [9] concluded that there was a need to undertake randomised controlled trials (RCT) to evaluate the cost-effectiveness of RASP in both primary and revisional bariatric procedures. This is the rationale behind the ongoing ROBOBAR study [10], a French academic-initiated RCT comparing the cost-effectiveness of the Da Vinci robot relative to laparoscopic bariatric surgery at 1 year.

Our study has several strengths, which we have emphasised, but it also has limitations.

We compared the mean costs of Da Vinci system medical devices per surgery against both the mean costs for medical consumables, corresponding to the same cost category within the ENC, and the cost of DRGs, assuming that the cost of Da Vinci system medical devices could serve as a proxy for mean costs of medical consumables as defined in the ENC. This approach represents a simplification of cost calculations. Indeed, outside Da Vinci devices, a variety of other medical devices, sterile or non-sterile, are routinely used during hospital stays, both in the operating room (insufflation ports, drapes, sutures, etc.) and in the subsequent management of patients on surgical wards (dressings, infusion lines, etc.). While most of these devices are inexpensive, some can be costly. For example, certain RASP, such as partial nephrectomies, lobectomies, or gynaecologic procedures, may occasionally use the AIRSEAL^®^ insufflation device for low-pressure pneumoperitoneum [11], adding substantial cost.

It is reasonable to estimate that the cost of non-Da Vinci system medical devices would increase total device costs by approximately 5–10%. Although we calculated total costs of Da Vinci system devices both with and without applying a discount rate, we focused primarily on undiscounted total costs. Given that a 5% discount rate was used, the inclusion of non-specific medical devices would place total costs within the same range as those reported here.

Similarly, our primary focus was on consumable medical devices, which means that acquisition and maintenance costs associated with the Da-Vinci system were not included in our base-case cost comparison.

Another limitation relates to our focus solely on the additional costs associated with robotic surgery, specifically the costs of medical devices, which means that potential cost savings from shorter LoS were not considered in our base-case cost comparison.

This is why we conducted an exploratory cost comparison incorporating additional medical resource use such as acquisition and maintenance costs, and potential gain associated with LoS difference. These analyses suggest that for some surgeries, the additional costs associated with Da-Vinci medical devices may be partially offset by cost reduction outside the operating room. Indeed, we observed that for the majority of surgeries, the mean LoS in our hospitals was numerically lower than national benchmarks. However, these results should be interpreted cautiously as it attributes a reduced LoS entirely to robotic surgery, which is a strong assumption. Indeed, other factors than the use of robotic surgery can influence the LoS. One such example is Enhanced Recovery After Surgery (ERAS) protocols [12] as they can have a significant organisational impact on patient stays.

Our results indicate potential avenues for future research.

Our method to calculate the cost of robotic-surgeries using the repository from the manufacturer could be expanded beyond our setting. For example, we assume that the systematic use of data from robots across all hospitals participating in the national costing study could improve the accuracy of data collection, which could more accurately evaluate the cost of DRGs associated with Da Vinci system surgeries and ultimately allow for better valuation of stays.

Moreover, such an approach could also strengthen cost-effectiveness evaluations of robotic-assisted surgery from the hospital perspective.

Overall, these results which are based on a focused cost-analysis, call for a more comprehensive economic evaluation that would include additional resource use during the index stay (operating room time, staff time) and outside the index stay (cost of readmissions / complications).

Last, updating our medical resource use and cost analysis should be considered to evaluate changes of practice and potential cost-reduction as the learning curve will be attained.

## Conclusion

For most surgical procedures, hospital funding based on current DRG tariffs is insufficient to cover the actual costs of robotic-assisted procedures, indicating that these stays are significantly undervalued even though a reduced LoS may partially offset additional costs. Urology is a notable exception where funding was found to be sufficient to cover procedural device costs.

## Supplementary Information

Below is the link to the electronic supplementary material.

Supplementary Material 1

## Data Availability

All available data are reported in the manuscript and in the supplementary information document.
