# Supplementary material for: Assessing the adequacy of funding for robotic-surgery hospital stays: a focused cost analysis based on 1,722 procedures in a French university hospital
Source: J Robot Surg. 2026 Apr 3;20(1):403. doi: 10.1007/s11701-026-03365-x (PMC13048915; doi:10.1007/s11701-026-03365-x)
Supplement: Supplementary file 1 — Supplementary Material 1 [file 11701_2026_3365_MOESM1_ESM.docx]

**Supplementary Information**

[Supplementary Table 1 : number and frequency of use of reusable DaVinci system instruments 2](#_Toc224503699)

[Supplementary Table 2 : number and frequency of use of single-use DaVinci system medical devices 3](#_Toc224503700)

[Supplementary Table 3: Comparison of costs (expressed as Euros) over time for procedures performed more than 50 times, except prostatectomy and partial nephrectomy 4](#_Toc224503701)

[Supplementary Table 4 : Exploratory cost analysis incorporating acquisition and maintenance costs for the Da Vinci system, and savings from reduced Length of stay, based on the most frequently performed surgeries (n>20) 5](#_Toc224503702)

[Supplementary Table 5 : Cost of DaVinci system medical devices, cost of medical consumables as measured within the ENC, costs and tariffs of DRGs, lenght of stay within the hospital, and national length of stay for procedures with fewer than 20 cases* 9](#_Toc224503703)

| Device  reference | Designation | Number  of use | Frequency of use |
| --- | --- | --- | --- |
| 470230 | Large clip applier | 202 | 11.7% |
| 470327 | Medium-large clip applier | 67 | 3.9% |
| 470401 | Small clip applier | 4 | 0.2% |
| 470179 | Monopolar curved scissors (Hot shears) | 1524 | 88.5% |
| 470183 | Permanent cautery hook | 95 | 5.5% |
| 471344 | Curved bipolar dissector | 5 | 0.3% |
| 470033 | Black diamond micro forceps | 9 | 0.5% |
| 470347 | Tip-Up fenestrated grasper | 268 | 15.6% |
| 471205 | Fenestrated bipolar forceps | 1640 | 95.2% |
| 471172 | Maryland bipolar forceps | 422 | 24.5% |
| 471049 | Cadiere forceps | 606 | 35.2% |
| 471093 | ProGrasp™ forceps | 491 | 28.5% |
| 470207 | Tenaculum forceps | 54 | 3.1% |
| 420006 | Large Needle Driver | 43 | 2.5% |
| 470194 | Mega™ needle driver | 305 | 17.7% |
| 471309 | Mega SutureCut™ needle driver | 53 | 3.1% |
| 471006 | Large needle driver | 1011 | 58.7% |
| 471296 | Large SutureCut needle driver | 257 | 14.9% |
| 470184 | Permanent cautery spatula | 27 | 1.6% |

# Supplementary Table 1 : number and frequency of use of reusable DaVinci system instruments

|  | Device  reference | Designation | Number  of use | Frequency of use |
| --- | --- | --- | --- | --- |
|  | 420273/ | Da Vinci Si drapes (arm drape + Column drape) | 23 | 1.3% |
|  | 470015/470341 | Da Vinci Xi drapes (arm drape + Column drape) | 1699 | 98.7% |
|  | 480545 | SureForm 45 curved-tip instrument | 199 | 11.6% |
|  | 480445 | SureForm 45 instrument | 3 | 0.2% |
|  | 480460 | SureForm 60 instrument | 273 | 15.9% |
|  | 480299 | EndoWrist suction irrigator* | 25 | 1.5% |
|  | 48345W | Reload, SureForm 45, 2.5 white | 470 | 27.3% |
|  | 48345B | Reload, SureForm 45, 3.5 blue | 90 | 5.2% |
|  | 48345T | Reload, SureForm 45, 4.6 black | 418 | 24.3% |
|  | 48345G | Reload, SureForm 45, 4.3 green | 407 | 23.6% |
|  | 48360W | Reload, SureForm 60, 2.5 white | 121 | 7.0% |
|  | 48360B | Reload, SureForm 60, 3.5 blue | 201 | 11.7% |
|  | 48360T | Reload, SureForm 60, 4.6 black | 271 | 15.7% |
|  | 48360G | Reload, SureForm 60, 4.3 green | 317 | 18.4% |
|  | 480440 | SynchroSeal | 116 | 6.7% |
|  | 480422 | Vessel Sealer Extend | 237 | 13.8% |

# Supplementary Table 2 : number and frequency of use of single-use DaVinci system medical devices

| Surgery | Period | n | Median | Interquartile range | | p value |
| --- | --- | --- | --- | --- | --- | --- |
|  |  |  |  | 25th | 75th |  |
| Thoracic lobectomy | 2023 semester 1 | 14 | 2,807 | 2,426 | 3,139 | 0.0816 |
|  | 2023 semester 2 | 43 | 2,743 | 2,481 | 3,092 |  |
|  | 2024 semester 1 | 51 | 3,050 | 2,676 | 3,494 |  |
| Hysterectomy | 2023 semester 1 | 44 | 1,354 | 1,237 | 1,354 | 0.7586 |
|  | 2023 semester 2 | 64 | 1,354 | 1,237 | 1,354 |  |
|  | 2024 semester 1 | 90 | 1,354 | 1,237 | 1,354 |  |
| Proctectomy | 2023 semester 1 | 23 | 1,841 | 1,841 | 2,395 | 0.1809 |
|  | 2023 semester 2 | 25 | 1,841 | 1,237 | 1,976 |  |
|  | 2024 semester 1 | 21 | 1,841 | 1,641 | 2,245 |  |
| Hepatectomy/ hepatic resection | 2023 semester 1 | 15 | 1,670 | 1,075 | 2,023 | 0.0474 |
|  | 2023 semester 2 | 24 | 1,924 | 1,558 | 2,331 |  |
|  | 2024 semester 1 | 29 | 2,072 | 1,851 | 2,460 |  |
| Myomectomy | 2023 semester 1 | 24 | 1,488 | 1,219 | 1,488 | 0.0549 |
|  | 2023 semester 2 | 16 | 1,488 | 1,488 | 1,510 |  |
|  | 2024 semester 1 | 25 | 1,488 | 1,378 | 1,531 |  |
| Thoracic segementectomy | 2023 semester 1 | 4 | 2,960 | 2,731 | 3,393 | 0.2957 |
|  | 2023 semester 2 | 16 | 2,780 | 2,607 | 3,256 |  |
|  | 2024 semester 1 | 32 | 3,262 | 2,656 | 3,979 |  |

# Supplementary Table 3: Comparison of costs (expressed as Euros) over time for procedures performed more than 50 times, except prostatectomy and partial nephrectomy

| Surgical specialty | Procedures | N | (1): Mean cost of DaVinci system medical devices | *(2): A*cquisition /maintenance costs per procedure | *(3): Benchmark costs (€),ie medical consumables* | Mean length of stay (days) | *Benchmark length of stay (days)* | *Difference in LoS* | *(4): Potential savings from reduced LoS* | Base case cost comparison: $\left( 1 \right)-(3)$ | Exploratory cost comparison  (1) + (2) +  (4) – (3) |
| --- | --- | --- | --- | --- | --- | --- | --- | --- | --- | --- | --- |
| Digestive surgery | Proctectomy | 69 | 1,854 | 704 | 2,346 | 10.43 | 15.11 | -4.68 | -4,680 | -492 | -4,468 |
|  | Hepatectomy/ hepatic resection | 68 | 1,950 | 704 | 1,921 | 7.75 | 10.47 | -2.72 | -2,720 | 29 | -1,987 |
|  | Colectomy | 48 | 2,086 | 704 | 1,123 | 6.31 | 8.59 | -2.28 | -2,280 | 963 | -613 |
|  | Sleeve Gastrectomy | 44 | 2,858 | 704 | 799 | 2.48 | 3.44 | -0.96 | -960 | 2,059 | 1,803 |
|  | Hernia cure | 40 | 1,235 | 704 | 429 | 2.45 | 1.92 | 0.53 | 530 | 806 | 2,040 |
|  | Rectopexy | 35 | 1,470 | 704 | 496 | 1.4 | 3.58 | -2.18 | -2,180 | 974 | -502 |
|  | Ventral hernia repair | 33 | 1,207 | 704 | 496 | 2.55 | 3.51 | -0.96 | -960 | 711 | 455 |
|  | Bypass/bipartition transit | 25 | 3,189 | 704 | 761 | 2.2 | 3.02 | -0.82 | -820 | 2,428 | 2,312 |
| Gynecology and breast surgery | Hysterectomy | 198 | 1,357 | 704 | 725 | 1.75 | 2.55 | -0.8 | -800 | 632 | 536 |
|  | Myomectomy | 65 | 1,455 | 704 | 547 | 1.34 | 2.51 | -1.17 | -1,170 | 908 | 442 |
|  | Deep infiltrating endometriosis surgery | 22 | 1,580 | 704 | 762 | 3.32 | 3.92 | -0.6 | -600 | 818 | 922 |
| Thoracic surgery | Lobectomy | 108 | 2,932 | 704 | 1,430 | 6.64 | 7.33 | -0.69 | -690 | 1,502 | 1,516 |
|  | Segmentectomy | 52 | 3,154 | 704 | 1,362 | 5.67 | 6.36 | -0.69 | -690 | 1,792 | 1,806 |
| Urology | Prostatectomy | 258 | 1,282 | 704 | 1,975 | 1.4 | 3.33 | -1.93 | -1,930 | -693 | -1,919 |
|  | Partial nephrectomy | 143 | 1,387 | 704 | 1,370 | 3.31 | 5.25 | -1.94 | -1,940 | 17 | -1,219 |
|  | Renovascular surgery | 37 | 1,488 | 704 | 776 | 3.97 | 4.73 | -0.76 | -760 | 712 | 656 |
|  | Cystectomy (+ bricker or ureterostomy) | 33 | 2,201 | 704 | 2,310 | 13.94 | 17.48 | -3.54 | -3,540 | -109 | -2,945 |
|  | Enlarged nephrectomy | 28 | 1,259 | 704 | 1,423 | 5.79 | 6.34 | -0.55 | -550 | -164 | -10 |
|  | Surgery for urological malformations | 26 | 1,397 | 704 | 1,088 | 2.4 | 4.04 | -1.64 | -1,640 | 309 | -627 |
|  | Surgery for urinary continence and pelvic organ prolapse | 22 | 1,334 | 704 | 543 | 2.18 | 3.25 | -1.07 | -1,070 | 791 | 425 |
|  | Cystectomy (+ bladder augmentation, neobladder, bladder replacement, ileal reservoir, or ileocecal bladder) | 21 | 1,621 | 704 | 2,347 | 15 | 18.66 | -3.66 | -3,660 | -726 | -3,682 |

# Supplementary Table 4 : Exploratory cost analysis incorporating acquisition and maintenance costs for the Da Vinci system, and savings from reduced Length of stay, based on the most frequently performed surgeries (n>20)

| Specialty | Procedures | N | Mean cost of DaVinci system medical devices (€) | *Benchmark costs (€),ie medical consumables* | Mean cost of DRG (€) | Mean Tariff of DRG(€) | Mean length of stay (days) | *Benchmark length of stay (days)* |
| --- | --- | --- | --- | --- | --- | --- | --- | --- |
| **Digestive surgery** | Bariatric revision | 19 | 2 967 | 1 037 | 8 118 | 7 164 | 4.26 | 7.08 |
|  | Cholecystectomy | 12 | 1 357 | 636 | 5 487 | 5 176 | 1.67 | 3.96 |
|  | Biliopancreatic diversion | 11 | 3 735 | 740 | 4 709 | 5 218 | 3.09 | 2.64 |
|  | Abdominoperineal resection/Total proctocolectomy | 10 | 2 283 | 2 576 | 21 269 | 17 240 | 12.9 | 16.34 |
|  | Oesophagectomy | 10 | 2 817 | 3 145 | 32 162 | 34 017 | 22.9 | 25.13 |
|  | Gastrectomy | 9 | 2 025 | 2 143 | 18 511 | 16 010 | 13.67 | 14.54 |
|  | Bypass takedown | 8 | 2 926 | 914 | 8 274 | 7 285 | 7.38 | 7.04 |
|  | Duodenopancreatectomy | 8 | 2 259 | 2 835 | 27 611 | 31 269 | 30.13 | 25.05 |
|  | Biliary tract surgery | 7 | 2 072 | 1 586 | 15 511 | 16 106 | 19.14 | 15.47 |
|  | Removal of prosthetic material | 7 | 1 421 | 780 | 6 780 | 5 971 | 7.14 | 6.6 |
|  | Colectomy + hepatic resection | 5 | 1 232 | 1 087 | 9 639 | 9 203 | 5 | 8.03 |
|  | Liver cyst resection | 5 | 1 714 | 1 124 | 8 545 | 9 034 | 3.4 | 5.4 |
|  | Hiatal hernia repair | 4 | 1 429 | 729 | 5 902 | 6 205 | 4 | 3.78 |
|  | Living donor left hepatectomy | 3 | 3 018 | 1 389 | 10 508 | 10 686 | 7.67 | 7.99 |
|  | Pancreatectomy | 3 | 2 574 | 1 855 | 15 751 | 14 716 | 10 | 12.19 |
|  | Exploratory coelioscopy | 2 | 2 006 | 864 | 5 600 | 5 384 | 2.5 | 3.11 |
|  | Colectomy + enlarged nephrectomy | 1 | 2 895 | 887 | 8 822 | 9 103 | 14 | 7,3 |
|  | Para-aortic lymph node dissection | 1 | 1 354 | 1 982 | 8 434 | 6 828 | 1 | 3.05 |
|  | Cytoreductive surgery | 1 | 1 641 | 1 220 | 13 504 | 10 713 | 7 | 12.86 |
|  | Partial gastrectomy + cholecystectomy | 1 | 1 234 | 991 | 7 728 | 7 091 | 2 | 4.42 |
|  | Hiatal hernia repair+bypass | 1 | 2 583 | 774 | 5 019 | 4 125 | 2 | 2.33 |
|  | Interaortocaval sampling | 1 | 1 393 | 578 | 4 056 | 4 563 | 2 | 2.66 |
|  | Lengthening of the biliary and alimentary limbs using the kissing-X technique | 1 | 3 579 | 810 | 6 864 | 6 701 | 2 | 4,33 |
|  | Rectal resection | 1 | 1 219 | 3 006 | 29 665 | 18 242 | 10 | 25,15 |
|  | Splenectomy | 1 | 1 849 | 793 | 8 180 | 8 265 | 5 | 7.56 |
| **Gynecology and breast surgery** | Complex deep infiltrating endometriosis surgery | 16 | 1 897 | 1 645 | 9 780 | 8 145 | 4.13 | 6.5 |
|  | Sacrocolpopexy | 15 | 1 328 | 596 | 4 597 | 4 178 | 1.47 | 2.29 |
|  | Mastectomy | 11 | 1 028 | 890 | 7 160 | 5 681 | 2.45 | 2.7 |
|  | Adnexectomy | 7 | 1 110 | 400 | 3 476 | 2 789 | 1.14 | 1.44 |
|  | Sacrocolpopexy and salpingectomy | 4 | 1 311 | 587 | 4 263 | 4 001 | 1.25 | 1.9 |
|  | Colpectomy | 3 | 1 478 | 622 | 5 892 | 4 469 | 4.67 | 4.86 |
|  | Exploratory laparoscopy | 2 | 1 293 | 346 | 3 229 | 2 689 | 1 | 0.92 |
|  | Lumbo-aortic lymph node dissection | 2 | 1 345 | 1 203 | 7 462 | 7 826 | 1 | 2.68 |
|  | Kystectomy + omentectomy | 2 | 1 376 | 420 | 3 503 | 3 265 | 1 | 0.48 |
|  | Salpingectomy | 2 | 1 548 | 367 | 3 203 | 2 383 | 1.5 | 0.86 |
|  | Uterine polypectomy | 2 | 1 815 | 672 | 9 165 | 7 450 | 4 | 8.15 |
|  | Accessory cavitated uterine mass resection | 1 | 1 354 | 547 | 4 135 | 3 054 | 1 | 2.51 |
|  | Adnexectomy and omentectomy | 1 | 1 085 | 378 | 3 457 | 2 771 | 1 | 1.77 |
|  | Change of breast prosthesis | 1 | 1 109 | 550 | 6 049 | 4 848 | 7 | 4.12 |
|  | Pelvic dissection | 1 | 1 378 | 1 203 | 7 462 | 7 826 | 2 | 2.68 |
|  | Partial cystectomy + hystero-reduction | 1 | 1 403 | 254 | 2 361 | 1 234 | 1 | 0.32 |
|  | Enlarged hysterectomy | 1 | 1 445 | 1 203 | 7 462 | 8 065 | 3 | 2.52 |
|  | Partial hysterectomy | 1 | 1 372 | 548 | 4 415 | 3 435 | 1 | 1,78 |
|  | Isthmocele repair | 1 | 1 403 | 547 | 4 135 | 2 855 | 1 | 2.65 |
|  | Kystectomy | 1 | 1 060 | 378 | 3 457 | 2 689 | 1 | 1.84 |
|  | Kystectomy + salpingectomy | 1 | 1 085 | 448 | 3 502 | 2 418 | 1 | 1.66 |
|  | Myomectomy + kystectomy | 1 | 1 262 | 547 | 4 135 | 3 054 | 1 | 2.51 |
|  | Myomectomy + digestive resection | 1 | 2 130 | Not available | Not available | 12 159 | 10 | 12.29 |
|  | Myomectomy + salpingectomy | 1 | 1 488 | 448 | 3 502 | 2 418 | 1 | 1.66 |
|  | Perineorrhaphy | 1 | 1 085 | 752 | 7 434 | 4 983 | 3 | 5.03 |
|  | Sacrocolpopexy and adnexectomy | 1 | 1 237 | 587 | 4 263 | 4 061 | 1 | 1.85 |
|  | Sacrocolpopexy, subtotal hysterectomy, and perineorrhaphy | 1 | 1 354 | 548 | 4 415 | 3 333 | 2 | 1,78 |
| **Pediatric surgery** | Ureteropelvic junction (pyeloplasty) | 8 | 1 328 | 881 | 5 708 | 4 400 | 1 | 3 |
|  | Cholecystectomy | 7 | 1 211 | 360 | 3 220 | 2 777 | 1 | 1.98 |
|  | Gastroesophageal Reflux Disease surgery | 6 | 1 238 | 389 | 5 162 | 5 251 | 2 | 3.76 |
|  | Total nephrectomy | 6 | 1 611 | 1 007 | 6 577 | 5 308 | 1.5 | 3.23 |
|  | Splenectomy | 3 | 1 631 | 644 | 5 411 | 5 707 | 2 | 3.51 |
|  | Bilio-digestive anastomosis | 2 | 1 996 | 735 | 7 007 | 10 730 | 4.5 | 4.16 |
|  | Nephrectomy | 2 | 1 410 | 1 082 | 7 389 | 6 394 | 1.5 | 3.03 |
|  | Partial nephrectomy | 2 | 1 413 | 1 261 | 8 806 | 8 220 | 12.5 | 3.58 |
|  | Nephro-ureterectomy | 2 | 1 014 | 969 | 6 171 | 4 693 | 1 | 3.21 |
|  | Thoracic segmentectomy | 2 | 1 682 | 1 552 | 13 463 | 14 316 | 9.5 | 9.81 |
|  | Splenectomy + cholecystectomy | 2 | 1 713 | 644 | 5 411 | 5 736 | 1.5 | 3.55 |
|  | Thymectomy | 2 | 1 038 | 822 | 6 041 | 6 172 | 2.5 | 3.08 |
|  | Hernia repair | 1 | 1 445 | 1 031 | 16 235 | 14 882 | 5 | 11.48 |
|  | Cystoscopy + Nephrectomy | 1 | 1 344 | 969 | 6 171 | 4 693 | 1 | 3.21 |
|  | Excision of müllerian residues | 1 | 950 | 548 | 4 415 | 3 435 | 2 | 1,78 |
|  | Partial hysterectomy | 1 | 1 109 | 548 | 4 415 | 3 333 | 2 | 1,78 |
|  | Surrenalectomy | 1 | 2 008 | 1 479 | 11 770 | 10 456 | 3 | 8.32 |
| **Thoracic** | Thymectomy | 9 | 902 | 1 127 | 9 187 | 8 711 | 4.11 | 6.65 |
|  | Mediastinal mass resection | 5 | 797 | 1 185 | 8 732 | 7 796 | 4 | 5.69 |
|  | Bronchogenic cyst resection | 4 | 1 160 | 1 052 | 7 198 | 7 184 | 2.5 | 4.19 |
|  | Intrathoracic lymph node biopsy | 3 | 748 | 1 000 | 7 280 | 6 626 | 4 | 4.38 |
|  | Wedge | 3 | 2 828 | 1 054 | 7 516 | 7 499 | 3 | 4.39 |
|  | Lymph node dissection | 1 | 1 050 | 991 | 7 728 | 7 091 | 2 | 4.42 |
|  | Zenker's diverticulum | 1 | 1 001 | 2 942 | 29 746 | 24 358 | 46 | 27.32 |
|  | Partial thoracic resection | 1 | 952 | 625 | 5 321 | 5 182 | 3 | 4,03 |
|  | Diaphragmatic hernia | 1 | 1 237 | 1 081 | 7 490 | 6 488 | 3 | 5.47 |
|  | Pleuropericardial cyst | 1 | 781 | 618 | 7 435 | 6 608 | 4 | 7,30 |
|  | Lewis Santy oesophagectomy | 1 | 3 847 | 4 204 | 42 976 | 41 535 | 24 | 32.26 |
| **Urology** | Nephro-ureterectomy | 15 | 1 428 | 1 918 | 15 542 | 12 539 | 5.27 | 10.92 |
|  | Other urologic cancer surgeries | 8 | 1 475 | 1 519 | 14 509 | 11 556 | 4.38 | 13.69 |
|  | Total nephrectomy | 3 | 1 235 | 1 208 | 8 060 | 6 962 | 8 | 5.43 |
|  | Partial cystectomy | 1 | 1 244 | 969 | 6 171 | 4 693 | 5 | 3.21 |

# Supplementary Table 5: Cost of DaVinci system medical devices, cost of medical consumables as measured within the ENC, costs and tariffs of DRGs, length of stay, and national length of stay for procedures with fewer than 20 cases*

* Head and Neck surgery was not investigated further due the limited number of procedures
